# Inferior patient-reported outcomes after total knee arthroplasty for post-traumatic versus primary osteoarthritis: a registry study

**DOI:** 10.1186/s12891-026-09875-x

**Published:** 2026-04-23

**Authors:** Ioannis Syrikas, Neel Desai, Georgios Tsikandylakis, Ioannis Karikis

**Affiliations:** 1 Department of Orthopaedics, NU-Hospital Group, Trollhättan/Uddevalla, Region of Västra Götaland Sweden; 2 Department of Orthopaedics, Institute of Clinical Sciences, Sahlgrenska Academy, University of Gothenburg, Gothenburg, Sweden; 3 Department of Orthopaedics, Sahlgrenska University Hospital, Gothenburg, Region of Västra Götaland Sweden; 4 Department of Research and Development, NU-Hospital Group, Trollhättan, Sweden; 5 Department of Orthopaedics, NU-Hospital Group, Uddevalla, 45180 Sweden

**Keywords:** TKA, PROM, Osteoarthritis, Knee, Fracture

## Abstract

**Background:**

Total knee arthroplasty (TKA) performed after knee fracture osteosynthesis for post-traumatic osteoarthritis (PTOF) is technically demanding and may yield inferior outcomes compared with TKA performed for primary osteoarthritis (OA). Comparing patient-reported outcomes (PROs) between these groups is challenging due to differing patient characteristics.

**Methods:**

This observational matched case–control study used data from the Swedish Arthroplasty Register and the Swedish Fracture Register and included 1293 TKAs performed for PTOF between 2000 and 2021, matched 1:2 with TKAs performed for OA based on sex, age, BMI, ASA classification, and time period. For each PTOF TKA and its matched controls, at least one of the following patient-reported outcome measures (PROMs) was available both preoperatively and 1-year postoperatively: KOOS-12, EQ-5D-3L, Likert pain and satisfaction, resulting in four separate matched PTOF cohorts depending on the available PROM.

**Results:**

Both groups demonstrated postoperative improvement. For the primary outcome, the PTOF group had a lower 1-year postoperative KOOS-12 total score than the OA group (56 vs 68, *p* < 0.001), and inferior scores were observed in all KOOS-12 subscales. Fewer patients in the PTOF group reported “no pain” (25% vs 41%, *p* = 0.003) or were “very satisfied” (43% vs 54%, *p* = 0.003) than those in the OA group. In linear regression analyses adjusted for type of articulation and corresponding preoperative PROM scores, PTOF remained associated with worse 1-year KOOS-12 outcomes and a lower EQ-5D-3L index score, whereas the adjusted difference in EQ-VAS was not statistically significant.

**Conclusions:**

Although PROs improved in both groups after TKA, PTOF patients reported inferior outcomes compared with OA patients. This difference is likely due to prior trauma and altered knee biomechanics, indicating that PTOF patients should be appropriately counselled preoperatively.

**Level of evidence:**

III.

**Type of study:**

Registry-based matched case–control study.

**Supplementary Information:**

The online version contains supplementary material available at 10.1186/s12891-026-09875-x.

## Background

Knee fractures are a well-documented and significant risk factor for developing post-traumatic osteoarthritis. It is estimated that 20–50% of patients with a history of trauma develop osteoarthritis, the prevalence of which is estimated to approximately 12% of all symptomatic knee osteoarthritis cases [1].

Total knee arthroplasty (TKA) is widely regarded as an effective surgical treatment for advanced osteoarthritis, providing pain relief and improved function in most cases [2, 3]. Over the recent years, the number of TKAs performed has increased significantly, making it one of the most common joint replacement surgeries [4, 5]. While TKA is highly effective for treating primary osteoarthritis, it may entail technical challenges and, in some cases, lead to less favourable outcomes when performed in patients with posttraumatic osteoarthritis after knee fracture osteosynthesis (PTOF) [6]. Several studies, comparing outcomes after TKA between patients with PTOF and those with primary osteoarthritis, have demonstrated higher complication rates and a tendency toward inferior patient-reported outcomes (PROs) in the former group. These studies, however, have been mostly conducted on small cohorts or within single institutions, limiting the generalizability of their findings [7–10]. Two recent systematic reviews [11, 12] underscored the higher complication rates and poorer PROs in patients undergoing TKA due to PTOF compared with those with primary osteoarthritis. Registry studies [13–15] have also indicated an elevated risk of early revision and complications in PTOF patients; however, no large-scale registry studies have been conducted to specifically compare these two patient groups in terms of postoperative PROs.

The current study utilized data from the Swedish Arthroplasty Register (SAR) and the Swedish Fracture Register (SFR) and aimed to compare PROs at 1 year after TKA, between PTOF patients and patients with primary knee osteoarthritis without any documented previous knee fracture or surgery (OA). We hypothesized that PTOF patients would have inferior PROs.

## Methods

### Study design

An observational register-based, matched cohort study was designed, merging data from the SAR and SFR. The main data source for identifying TKAs performed for PTOF and OA was the SAR. The data were then linked to the SFR to potentially identify PTOF cases whose previous osteosynthesis had not been reported to the SAR. The study was conducted in accordance with the Strengthening the Reporting of Observational Studies in Epidemiology (STROBE) guidelines, ensuring a rigorous and transparent approach to observational research [16].

### Setting

The SAR, a merger of the Swedish Knee and Hip Arthroplasty Registers, has collected data on knee replacements since 1975. Reporting is mandatory for all units performing knee prostheses procedures, with coverage exceeding 97%. The SAR provides detailed information on patients, surgical techniques, implant types, reoperations, revisions, as well as patient-reported outcome measures (PROMs) and can be linked with other national registries using the Swedish personal identification number for comprehensive data analysis [17]. Previous osteosynthesis around the knee is recorded in the SAR as a binary variable, without any further information such as type of fracture, location or type of osteosynthesis. The SAR also provides information on other previous knee surgery, such as osteotomy around the knee, arthroscopic surgery, meniscal and anterior cruciate ligament surgery. A nationwide PROM program had not been established in the Swedish Knee Arthroplasty Register (SKAR) until the merger with the Swedish Hip Arthroplasty Register in 2021. Until then, PRO instruments differed across hospitals. The most common PROMs recorded in the SKAR included the 12-item Knee injury and Osteoarthritis Outcome Score (KOOS-12), the 3 level 5-dimensional Euroquol questionnaire (EQ-5D-3L) and knee pain and patient satisfaction assessed on a 5-point Likert scale.

The SFR is a web-based, nationwide registry that prospectively collects detailed data on orthopaedic fractures. Inclusion requires a permanent Swedish personal identification number, and only fractures occurring within Sweden are recorded. Data collection began in 2011, with 72% of orthopaedic departments participating by 2016. By 2021, all orthopaedic clinics in Sweden were mandated to report fractures, achieving 100% national coverage [18].

### Participants

**Fig. 1:**
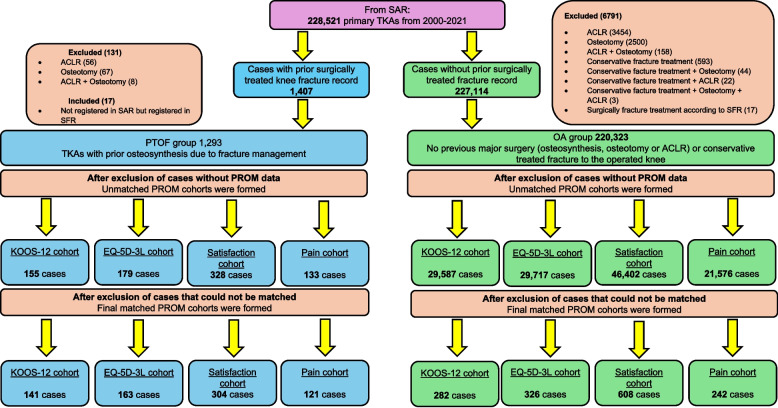
Flowchart of TKAs included in the study (PTOF vs OA groups). *TKAs,* total knee arthroplasties*; PTOF,* posttraumatic osteoarthritis after knee fracture osteosynthesis*; OA,* knee osteoarthritis without prior significant knee surgery or fractures*;* KOOS-12, a 12-item short form of the Knee injury and Osteoarthritis Outcome Score; EQ-5D-3L, the 3-level version of EQ-5D – a standardized measure of health-related quality of life developed by the EuroQol Group; *ACLR*, anterior cruciate ligament reconstruction; *SAR*, Swedish Arthroplasty Register; *SFR*, Swedish Fracture Register; *PROM*, patient-reported outcome measures

The study population included two groups of TKA patients: PTOF and OA. PTOF included adult patients from the SAR who had undergone TKA between 2000–2021 and had previously sustained a fracture around the knee treated with osteosynthesis according to the binary (yes/no) variable but no other previous knee surgery recorded in the SAR. Because previous osteosynthesis might be underreported in the SAR, due to the usually long time interval between knee fracture and the development of posttraumatic osteoarthritis requiring TKA, data from the SAR were linked to the SFR for the period 2011–2018. The linkage resulted in 17 more cases in the PTOF group who had unregistered previous knee osteosynthesis in the SAR but had a confirmed osteosynthesis in the SFR prior to TKA. The reason for the low contribution of the SFR to the PTOF group could be its low coverage during the period 2011–2016 (< 72%). After the linkage, 1293 PTOF TKAs were identified. Among them, there were different subsets of patients with PROM data on KOOS-12, EQ-5D-3L, satisfaction and pain, as well as patients with no recorded PROMs. Only cases with both pre- and postoperative PROM data were included in the study, except for satisfaction, which is solely reported postoperatively, resulting in four separate PTOF cohorts, each consisting of 133 to 328 TKAs (Fig. 1). These cohorts represent outcome-specific subsets of the same underlying PTOF population and therefore partially overlap. (Supplementary Table S3).

The OA group served as the control group and consisted of patients extracted from the SAR who had undergone TKA due to primary knee osteoarthritis without any prior fracture or surgery around the same knee recorded in either register. After exclusion of OA cases lacking recorded PROMs, four OA cohorts (one for each PROM) were created, comprising between 21576 to 46402 in each cohort (Fig. 1).

### Study variables and outcome

The data extracted from the SAR included patient age, sex, body mass index (BMI) and comorbidity in terms of American Society of Anesthesiologists (ASA) classification at index TKA, operated side, and surgery date. Furthermore, the indications of surgery, type of knee articulation (cruciate retaining (CR), posterior stabilized (PS), constrained condylar knee (CCK) or hinged), previous surgeries around the knee joint, PROM data preoperatively and at 1-year postoperatively were retrieved. The PROMs included KOOS-12 (0–100, where 100 denoted the best outcome), the EQ-5D-3L index (0–1, where 1 denoted full health while 0 a health state equivalent to death), EQ-VAS (0–100, where 100 denoted the best possible self-reported health status), a patient satisfaction Likert scale (1–5, where 5 denoted the best outcome), and a pain Likert scale (1–5 where 5 denoted severe pain). The primary outcome was the KOOS-12 total score at 1-year postoperatively. Secondary outcomes were health-related quality of life (HRQoL) at 1-year, measured with the EQ-5D-3L index and EQ-VAS, as well as patient satisfaction and pain measured on Likert scales. Between 2020 and 2021, following the merger of the two arthroplasty registries in Sweden, the new five-level version of the EQ-5D instrument (EQ-5D-5L) was introduced for use in elective TKAs. Therefore, the cohort for the EQ-5D-3L included cases only up to 2020, unlike the other PROMs analysed, which were collected until 2021. Because PROM collection was not harmonized across units and time periods, these outcomes were available in partially overlapping PROM-specific cohorts (Supplementary Table S3) and were therefore analysed within their respective matched cohorts.

Regarding the SFR, the extracted variables of interest for the purposes of the study included each patient's pseudonym and operated side for fracture treatment, type of fracture, date of fracture management or surgery, and type of osteosynthesis.

The 1-year postoperative interval was chosen as the primary endpoint since it aligns with the standardized follow-up period in the SAR and reflects the stage at which functional recovery and symptom relief after TKA typically plateau, with only minimal subsequent improvement [19].

### Matching

To ensure directly comparable groups and minimize bias, TKAs performed for PTOF were matched to those performed for OA based on age, sex, BMI, ASA classification, as well as the time period of the TKA procedure (≤ 2015 or ≥ 2016). The choice of 2015 as the cut-off was based on the substantial improvement in PROM coverage and reporting consistency in SKAR from that year onward, when the register began meeting internationally recommended standards of completeness [17, 20].

A 1:2 case–control matching was performed for the variables mentioned above. Exact matching was applied for sex, ASA classification, and time period, while a difference of ± 1 unit for BMI and ± 2 years for age was accepted. The 1:2 matching ratio was adopted to optimize statistical efficiency and maintain comparability between groups [21].

The quality of matching was evaluated descriptively by comparing baseline characteristics between groups before (Supplementary Table S1) and after matching (Table 1). Covariate balance after matching was assessed using standardized differences: standardized mean differences (SMDs) for continuous variables and standardized differences of proportions for categorical variables. The standardized differences for the matched variables were below 0.1 indicating acceptable balance [22].

**Table 1:** Demographic characteristics of matched PROM cohorts

Factor	Satisfaction Likert scale		Std diff	Pain Likert scale		Std diff	KOOS-12		Std diff	EQ-5D-3L		Std diff
	**PTOF group**	**OA** **group**		**PTOF group**	**OA** **group**		**PTOF group**	**OA** **group**		**PTOF group**	**OA** **group**	
Number of cases	304	608		121	242		141	282		163	326	
Age Mean (SD)	66.9 (8.8)	67.5 (8.7)	0.069	66.8 (9)	67 (9.1)	0.022	67.3 (8.6)	67.6 (8.5)	0.035	67.2 (9.1)	67.4 (9.1)	0.022
BMI Mean (SD)	28.1 (4.1)	28.1 (4.1)	0	27.6 (3.5)	27.7 (3.5)	0.029	28.2 (3.9)	28.2 (3.9)	0	28.1 (4.3)	28.1 (4.2)	0
Sex n (%)												
Male	161 (53)	322 (53)	0	72 (59)	144 (59)	0	78 (55)	156 (55)	0	82 (50)	164 (50)	0
Female	143 (47)	286 (47)	0	49 (41)	98 (41)	0	63 (45)	126 (45)	0	81 (50)	162 (50)	0
Side n (*%*)												
Right	160 (53)	294 (48)	0.086	102 (85)	186 (76)	0.189	65 (47)	146 (52)	0.114	79 (48)	158 (49)	0
Left	144 (47)	314 (50)	0.086	19 (15)	56 (23)	0.189	76 (53)	136 (48)	0.114	84 (52)	168 (51)	0
ASA classification n (*%*)												
1	54 (18)	108 (18)	0	17 (14)	34 (14)	0	24 (17)	48 (17)	0	31 (20)	62 (20)	0
2	198 (65)	396 (65)	0	84 (70)	168 (70)	0	93 (66)	186 (66)	0	102 (62)	204 (62)	0
3	52 (17)	104 (17)	0	20 (16)	40 (16)	0	24 (17)	48 (17)	0	30 (18)	60 (18)	0
4	0 (0)	0 (0)	0	0 (0)	0 (0)	0	0 (0)	0 (0)	0	0 (0)	0 (0)	0
5	0 (0)	0 (0)	0	0 (0)	0 (0)	0	0 (0)	0 (0)	0	0 (0)	0 (0)	0
Type of articulation n (*%*)												
CR	242 (79)	572 (93)	0.438	100 (82)	230 (95)	0.402	117 (83)	260 (92)	0.282	137 (84)	312 (95)	0.394
PS	43 (14)	22 (4)	0.377	17 (14)	12 (5)	0.314	15 (10)	21 (8)	0.111	19 (11)	11 (4)	0.318
CCK	17 (6)	10 (2)	0.213	4 (4)	0 (0)	0.261	9 (7)	1 (0)	0.339	7 (5)	3 (1)	0.213
Hinged	2 (1)	4 (1)	0	0 (0)	0 (0)	0	0 (0)	0 (0)	0	0 (0)	0 (0)	0
Year of TKA n (*%*)												
2000–2015	68 (23)	136 (23)	0	26 (22)	52 (22)	0	52 (37)	104 (37)	0	60 (37)	120 (37)	0
2016–2021	236 (77)	472 (77)	0	95 (78)	190 (78)	0	89 (63)	178 (63)	0	103 (63)	206 (63)	0

Data are presented as mean (standard deviation) or n (%). *BMI,* body mass index*; ASA,* American Society of Anesthesiologists*; PTOF,* posttraumatic osteoarthritis after knee fracture osteosynthesis*; OA,* knee osteoarthritis without prior significant knee surgery or fractures*; TKA,* total knee arthroplasty; *KOOS-12*, a 12-item short form of the Knee injury and Osteoarthritis Outcome Score; *EQ-5D-3L*, the 3-level version of EQ-5D is a standardised measure of health-related quality of life developed by the EuroQol Group; *CR*, cruciate retaining; *PS*, posterior stabilized; *CCK*, constrained condylar knee; *Std diff*, standardized difference (standardized mean difference [SMD] for continuous variables and standardized difference of proportions for categorical variables). Lower absolute values indicate better balance; values below 0.1 were considered acceptable

### Statistical analysis

Continuous variables were reported as means with their standard deviations (SD), and categorical variables as absolute and relative frequencies. Statistical analysis was performed using IBM SPSS Statistics version 29.0. All analyses were performed using available data, without imputation for missing values. Cases with missing values of matching or outcome variables were excluded during the patient selection process (Fig. 1). The statistical significance of differences between PTOF and OA in categorical PROMs (satisfaction and pain Likert scales) was tested using the chi-square test. For numerical PROMs (KOOS-12 and EQ-5D-3L), differences between groups were tested with the Mann–Whitney U-test, because the Kolmogorov–Smirnov test indicated a non-normal distribution for these variables. KOOS-12 was analysed as a total score (primary knee-specific outcome) and by its three subscales (pain, function, and knee-related quality of life) to facilitate domain-specific interpretation. Subscale analyses were considered supportive of the KOOS-12 total score and were interpreted in conjunction with effect sizes and 95% confidence intervals rather than as independent hypothesis tests. In addition, linear regression models were used to assess the association between study group and 1-year postoperative KOOS-12 and EQ-5D-3L outcomes. Each model included study group (PTOF vs OA), type of articulation, and the corresponding preoperative PROM score as covariates. Accordingly, postoperative KOOS-12 Pain, Function, Quality of life, and Total scores were adjusted for the corresponding preoperative KOOS-12 values, whereas postoperative EQ-5D-3L index and EQ-VAS were adjusted for the corresponding preoperative EQ-5D-3L values. Matching variables (age, sex, BMI, ASA classification, and year of surgery) were not additionally included in the regression models, as balance in these covariates had been achieved through the matching procedure. For ordinal outcomes (satisfaction and pain Likert scales), analyses were stratified by type of articulation, and chi-square or Fisher’s exact tests were applied as appropriate. The significance level was set at 0.05.

### Study size

The study included all PTOF cases, and their OA matches with available PROMs in SAR (Fig. 1). No sample size calculation was conducted.

## Results

### KOOS-12

The KOOS-12 cohort included 141 TKAs in the PTOF group, matched with 282 TKAs in the OA group (Table 2). At 1 year, total KOOS-12 score was 56 for PTOF and 68 for OA (*p* < 0.001). Statistically significantly inferior scores in all subscales as well as in the total KOOS-12 scale were reported by the PTOF group, both pre- and postoperatively. Adjusting for preoperative KOOS-12 score and type of articulation did not change the results, as postoperative total KOOS-12 score remained inferior in the PTOF group by 9.4 points (*p* < 0.001, Table 3).

**Table 2:** Preoperative and 1-year postoperative KOOS-12 subscale and total scores

Pre- and postoperative KOOS-12 scores				
**KOOS-12 scores**	**PTOF group** **(*n* = 141)**	**OA group** **(*n* = 282)**	**Mean Difference** **(CI)**	*p*-value*
	**Mean (SD)**	**Mean (SD)**		
Preoperative				
Pain subscale	31 (13)	36 (13)	−5 (−8.3 to −1.7)	** < 0.001**
Function subscale	30 (14)	37 (15)	−7 (−10.6 to −3.4)	** < 0.001**
Quality of life subscale	18 (12)	28 (12)	−10 (−12.8 to −7.2)	** < 0.001**
Total score	26 (11)	34 (12)	−8 (−10.6 to −5.4)	** < 0.001**
1-year postoperative				
Pain subscale	62 (18)	75 (8.3)	−13 (−16.5 to −9.5)	** < 0.001**
Function subscale	56 (18)	67 (13)	−11 (−14.8 to −7.2)	** < 0.001**
Quality of life subscale	50 (21)	63 (16)	−13 (−17.2 to −8.8)	** < 0.001**
Total score	56 (18)	68 (11)	−12 (−15.5 to −8.5)	** < 0.001**

Bold values indicate statistically significant differences (*p* < 0.05)

*SD* standard deviation, *PTOF* posttraumatic osteoarthritis after knee fracture osteosynthesis*, OA* knee osteoarthritis without prior significant knee surgery or fractures*, KOOS-12* a 12-item short form of the Knee injury and Osteoarthritis Outcome Score, *CI 95%* confidence intervals

^*^Mann–Whitney *U-test*

**Table 3:** Linear regression analysis of 1-year postoperative KOOS-12 and EQ-5D-3L outcomes, adjusted for type of articulation and the corresponding preoperative PROM scores

Linear regression of 1-year postoperative KOOS-12 scores adjusted for type of articulation and the corresponding preoperative KOOS-12 scores			
	**Adjusted coefficient for PTOF group (vs OA)**	**95% confidence intervals**	***p*-value**
Subscale: Pain	−12.4	−14.9 to −9.9	** < 0.001**
Subscale: Function	−7.7	−10.6 to −4.7	** < 0.001**
Subscale: Quality of life	−8.6	−12.4 to −4.9	** < 0.001**
Total score	−9.4	−12.2 to −6.7	** < 0.001**
Linear regression of 1-year postoperative EQ-5D-3L index and EQ-VAS scores adjusted for type of articulation and the corresponding preoperative EQ-5D-3L scores			
EQ-5D-3L index	−0.025	−0.044 to −0.005	**0.012**
EQ-VAS	−3.081	−6.50 to 0.34	0.077

Bold values indicate statistically significant differences (*p* < 0.05)

Linear regression models included study group (PTOF vs OA), type of articulation, and the corresponding preoperative PROM score as covariates. Thus, postoperative KOOS-12 outcomes were adjusted for preoperative KOOS-12 scores, whereas postoperative EQ-5D-3L outcomes were adjusted for the corresponding preoperative EQ-5D-3L index or EQ-VAS score. Matching variables (age, sex, BMI, ASA class, and year of surgery) were not additionally included, as balance in these factors was achieved through the matching procedure. *KOOS-12*, a 12-item short form of the Knee injury and Osteoarthritis Outcome Score*; EQ-5D-3L*, the 3-level version of EQ-5D is a standardised measure of health-related quality of life developed by the EuroQol Group; *EQ-VAS*, EuroQol visual analogue scale

### EQ-5D-3L

This cohort included 163 TKAs in the PTOF group, matched with 326 TKAs in the OA group. The EQ-5D-3L index and EQ-VAS scores did not show statistically significant differences between the two groups preoperatively. Postoperatively, PTOF patients reported lower scores in both these parameters than OA patients, with their differences, although small at group level, reaching statistical significance (Table 4). Adjusting for preoperative values of EQ5D-3L index or EQ-VAS respectively and type of articulation did not change the results for the postoperative EQ5D-3L index, but the difference in EQ-VAS did not reach statistical significance (Table 3).

**Table 4:** Preoperative and 1-year postoperative EQ-5D-3L index and EQ-VAS scores

Pre- and postoperative EQ-5D-3L scores				
	**PTOF group** **(*n* = 163)**	**OA group** **(*n* = 326)**	**Mean Difference** **(CI)**	*p*-value*
	**Mean (SD)**	**Mean (SD)**		
*Preoperative*				
EQ-5D-3L index	0.765 (0.1)	0.770 (0.1)	−0.005 (−0.026 to 0.016)	0.7
EQ-VAS	63 (22)	64 (23)	−1 (−6 to 4)	0.55
*1-year postoperative*				
EQ-5D-3L index	0.860 (0.1)	0.888 (0.1)	−0.028 (−0.048 to −0.008)	**0.008**
EQ-VAS	73 (20)	78 (18)	−5 (−9 to −1)	**0.04**

Bold values indicate statistically significant differences (*p* < 0.05)

*SD* standard deviation, *PTOF* posttraumatic osteoarthritis after knee fracture osteosynthesis, *OA* knee osteoarthritis without prior significant knee surgery or fractures, *EQ-5D-3L* the 3-level version of EQ-5D is a standardised measure of health-related quality of life developed by the EuroQol Group*, EQ-VAS* EuroQol visual analogue scale*, CI 95%* confidence intervals

^*^P-values were obtained using the Mann–Whitney U-test and adjusted using the Benjamini–Hochberg procedure

### Patient satisfaction

This cohort included 304 TKAs in the PTOF group, matched with 608 TKAs in the OA group (Table 5). The results revealed a statistically significant difference 1-year postoperatively between the two groups favoring TKA after OA. The proportions of satisfied patients were comparable, however a significantly higher proportion of PTOF patients were dissatisfied or very dissatisfied and a lower proportion of PTOF patients were very satisfied compared with OA (43% vs 54%, p = 0.003).

**Table 5:** Satisfaction likert scale results at 1-year postoperative follow-up

	**PTOF group**		**OA group**	
	**Number (*%*)**	**CI**	**Number (*%*)**	**CI**
1. Very dissatisfied	9 (3.1)	1.6 to 5.5	8 (1.3)	0.7 to 2.6
2. Dissatisfied	24 (7.9)	5.4 to 11.5	26 (4.3)	2.9 to 6.2
3. Neither nor	42 (14)	10.4 to 18.1	57 (9.4)	7.3 to 12
4. Satisfied	98 (32)	27.2 to 37.7	190 (31)	27.7 to 35
5. Very satisfied	131 (43)	37.6 to 48.7	327 (54)	49.8 to 57.7
Total	304 (100)		608 (100)	

Chi-square test (*p* = 0.003)

*PTOF* posttraumatic osteoarthritis after knee fracture osteosynthesis*, OA* knee osteoarthritis without prior significant knee surgery or fractures, *CI* 95% confidence intervals of proportions

### Pain

This cohort included 121 TKAs in the PTOF group, matched with 242 TKAs in the OA group (Table 6). No statistically significant difference was found between the two groups preoperatively. Postoperatively, the difference reached statistical significance, favoring the OA group, with considerably higher proportions of PTOF patients reporting mild to severe pain and a lower proportion of PTOF patients reporting "no pain" after the surgery compared with OA (25% vs 41%, *p* = 0.003).

**Table 6:** Preoperative and 1-year postoperative pain likert scale results

	**Preoperatively**				**1-year postoperatively**			
	**PTOF group**		**OA group**		**PTOF group**		**OA group**	
	**Number (*%*)**	**CI**	**Number (*%*)**	**CI**	**Number (*%*)**	**CI**	**Number (*%*)**	**CI**
1. None	0 (*0*)	-	0 (*0*)	-	30 (*25*)	18 to 33.2	100 (*41*)	35.3 to 47.6
2. Very mild	1 (*0.8*)	0.1 to 4.5	2 (*0.8*)	0.2 to 3	34 (*28*)	20.9 to 36.7	75 (*31*)	25.5 to 37.1
3. Mild	9 (*7.4*)	4 to 13.5	16 (*6.6*)	4.1 to 10.5	30 (*25*)	18 to 33.2	36 (*15*)	10.9 to 19.9
4. Moderate	54 (*45*)	36.1 to 53.5	124 (*51*)	45 to 57.5	19 (*15*)	10.3 to 23.2	25 (*10*)	7.1 to 14.8
5. Severe	57 (*47*)	38.4 to 56	100 (*41*)	35.3 to 47.6	8 (*6.6*)	3.4 to 12.5	6 (*2.5*)	1.1 to 5.3
Total	121 (*100*)		242 (*100*)		121 (*100*)		242 (*100*)	

Chi-square test: preoperatively (*p* = 0.7), 1-year postoperatively (*p* = 0.003)

*PTOF* posttraumatic osteoarthritis after knee fracture osteosynthesis*, OA* knee osteoarthritis without prior significant knee surgery or fractures, *CI* 95% confidence intervals of proportions

### Stratified analysis by type of articulation

In analyses stratified by type of articulation, patient satisfaction and knee pain remained statistically significantly different in favour of OA patients in CR TKA, whereas no statistically significant differences were observed in more constrained articulations (Supplementary Table S2). For Likert pain, the stratified analysis included CR and PS TKA only, because of the absence of CCK TKA in the OA group and hinged TKA in both groups.

## Discussion

This study aimed to compare 1-year post-TKA PROs between PTOF and OA patients. The present findings consistently favored the OA group across all four PROMs, including KOOS-12, the EQ-5D-3L and Likert scales for pain and patient satisfaction. Although both groups experienced improvements in PROs after TKA, PTOF patients reported lower knee function, lower satisfaction, higher levels of postoperative pain. HRQoL was also lower for PTOF patients, but the difference was small.

The added value of this study lies in its nationwide, registry-based design with linked SAR—SFR data and PROM-specific matching, enabling comparisons based on both preoperative and 1-year postoperative PROMs. The inclusion of both knee-specific and generic outcome measures was intended to strengthen the interpretability and clinical relevance of the results.

Previous studies showed that patients undergoing TKA for post-traumatic osteoarthritis after tibial plateau fracture experience higher complication rates, more reoperations, and inferior PROMs compared with primary OA [10, 11].

In the present study, inferior postoperative KOOS-12 outcomes in the PTOF group were observed both before and after adjustment for type of articulation and corresponding preoperative KOOS-12 scores. This suggests that the worse 1-year knee-specific outcomes in PTOF were not explained solely by lower baseline PROM levels and supports the interpretation that PTOF itself is associated with inferior postoperative knee-related outcomes.

Although PTOF patients reported lower postoperative EQ-5D-3L scores than OA patients, the between-group differences were smaller than those observed for the knee-specific outcomes. After adjustment for type of articulation and corresponding preoperative EQ-5D-3L scores, a significant difference remained for the EQ-5D-3L index, whereas no statistically significant difference was observed for EQ-VAS. This may indicate that generic HRQoL measures are less sensitive than knee-specific instruments in detecting differences related to knee function and symptoms.

For categorical outcomes such as satisfaction and pain, significant differences persisted in CR TKA, whereas statistically significant differences could not be demonstrated in more constrained TKA, possibly due to the smaller number of observations.

Poorer PROs in PTOF patients may reflect both the sequelae of the initial trauma and the increased technical complexity of TKA in PTOF. In contrast to primary osteoarthritis, PTOF often presents with localized cartilage loss, malalignment, and compromised soft-tissue balance, which may predispose to residual symptoms after reconstruction. Prior fracture fixation and subsequent procedures may also result in scar tissue and capsular adhesions, altered soft-tissue envelope, and limited motion, thereby contributing to postoperative stiffness and pain [7, 8, 12]. Importantly, TKA in PTOF is more often challenged by distorted anatomy, bone loss or metaphyseal defects, and ligamentous instability or insufficiency, which may necessitate additional releases, augmentation, stems, or higher levels of implant constraint to achieve stability [23, 24]. The higher use of constrained articulations observed in the PTOF cohort in the present study likely reflects these intraoperative requirements and may, in part, contribute to inferior functional recovery compared with primary OA.

In this context, the loss of statistical significance for EQ-VAS after adjustment for articulation type and preoperative EQ-VAS may suggest that differences in overall self-rated health are partly related to the greater use of constrained implants and the increased surgical complexity in PTOF patients. However, the smaller number of patients in the non-CR groups may also have reduced the precision of this analysis. The persistence of differences in KOOS-12 suggests that PTOF-related factors beyond implant constraint, such as soft-tissue scarring, stiffness, and residual malalignment, are also likely to contribute to inferior PROMs.

### Limitations

This study has several limitations. First, the follow-up period was restricted to 1 year, making it difficult to assess the long-term outcomes of TKA in PTOF patients. However, according to a recent systematic review and meta-analysis, long-term PROMs beyond one to two years often show minimal changes and incomplete data, raising questions about their meaningfulness. Therefore, a 1-year follow-up could still provide clinically relevant information [19].

While key demographic variables were matched between groups, unmeasured confounding like the experience and surgical volume of the surgeon may have affected the results. Since TKA on PTOF is considered a more difficult operation than OA TKA, it is plausible that PTOF patients have been operated by fewer and more skilled surgeons, while OA patients may have been operated by a mix of surgeons with varying experience and surgical volume. If the same set of surgeons had operated both groups, then the difference in PROMs may have been even greater. The present study could not account for surgeon volume and experience since these parameters are not registered in SAR.

Another limitation of the study is that the four PROMs derived from 4 separate patient cohorts rather than a single cohort, reducing data consistency. PROM data were missing systematically because the national PROM programme was not fully uniform during the study years. This pattern of missingness may have influenced the estimates to some extent and should be considered when interpreting the results. Additionally, the SFR was established in 2011; therefore, fractures and osteosynthesis procedures performed before 2011 could not be systematically verified through linkage with SFR data. This creates a risk of exposure misclassification during the early years of the study period and the transition to more complete fracture capture, particularly for older fractures treated with osteosynthesis where hardware may later have been removed. Consequently, some patients with a history of knee fracture fixation may not have been identified and could have been classified as controls.

Finally, due to the above-mentioned limitation, detailed data on fracture location (tibia, femur, patella) and type of osteosynthesis were available only in a restricted subset of PTOF patients, as this information is recorded in the SFR. Consequently, a meaningful subgroup PROM analysis by fracture site and fixation type was not feasible. This is clinically relevant because post-traumatic pathways following injuries around the knee are not uniform; for example, TKA following tibial plateau fractures has been associated with distinct reconstructive demands and outcome profiles compared with other periarticular injuries, and fracture site may therefore contribute to heterogeneity in prognosis [7, 13, 25]. Accordingly, our findings should be interpreted as an overall estimate for a mixed post-traumatic cohort and should not be taken to imply an identical prognosis across all PTOF subtypes.

## Conclusions

PTOF patients experienced decreased knee function, lower satisfaction, higher levels of pain, and had a tendency towards decreased HRQoL 1-year after TKA compared with OA patients. These findings highlight the challenges of treating PTOF patients, probably arising from the complex nature of joint damage following trauma. Thus, it is important to inform PTOF patients that the outcome after TKA may be less favourable than in patients undergoing the same procedure due to OA.

## Supplementary Information

Supplementary Material 1.

## Data Availability

The datasets generated and/or analysed during the current study are not publicly available due to Swedish data protection regulations and registry governance policies but are available from the corresponding author on reasonable request and subject to approval by the Centre of Registers Västra Götaland.

## Abbreviations

ACLR: Anterior cruciate ligament reconstruction
ASA: American Society of Anesthesiologists Classification
BMI: Body mass index
CI: Confidence intervals
CCK: Constrained condylar knee
CR: Cruciate retaining
EQ-5D-3L: EuroQol 5-Dimension 3-Level
EQ-5D-5L: EuroQol 5-Dimension 5-Level
HRQoL: Health-related quality of life
KOOS-12: Knee Injury and Osteoarthritis Outcome Score-12
OA: Knee osteoarthritis without prior significant knee surgery or fractures
PRO: Patient-reported outcome
PROM: Patient-reported outcome measure
PS: Posterior stabilized
PTOF: Posttraumatic osteoarthritic after knee fracture osteosynthesis
SAR: Swedish Arthroplasty Register
SD: Standard deviation
SFR: Swedish Fracture Register
SMD: Standardized mean difference
STROBE: Strengthening the Reporting of Observational Studies in Epidemiology
TKA: Total knee arthroplasty
VAS: Visual analogue scale
