# Supplementary material for: Inferior patient-reported outcomes after total knee arthroplasty for post-traumatic versus primary osteoarthritis: a registry study
Source: BMC Musculoskelet Disord. 2026 Apr 23;27:351. doi: 10.1186/s12891-026-09875-x (PMC13104453; doi:10.1186/s12891-026-09875-x)
Supplement: Supplementary file 1 — Supplementary Material 1. [file 12891_2026_9875_MOESM1_ESM.docx]

| **Supplementary Table S1. Demographic characteristics of unmatched PROMs cohorts** | | | | | | | | | | | |
| --- | --- | --- | --- | --- | --- | --- | --- | --- | --- | --- | --- |
| **Factor** | **Satisfaction Likert scale** | | | **Pain Likert scale** | | | **KOOS-12** | | | **EQ-5D-3L** | |
|  | **PTOF group** | **OA group** | **PTOF group** | | **OA group** | **PTOF group** | | **OA group** | **PTOF group** | | **OA group** |
| **Number of cases** | 328 | 46402 | 133 | | 21576 | 155 | | 29587 | 179 | | 29717 |
| **Age**  Mean (SD) | 66.4 (9.7) | 70 (8.5) | 65.8 (9.8) | | 69.8 (8.5) | 67 (9.6) | | 69.8 (8.4) | 66.7 (10.1) | | 69.9 (8.4) |
| **BMI**  Mean (SD) | 28.1 (4.5) | 28.7 (4.3) | 27.5 (3.9) | | 28.7 (4.3) | 28.3 (4.3) | | 28.8 (4.3) | 28.2 (4.6) | | 28.7 (4.3) |
| **Sex** n (%) |  |  |  | |  |  | |  |  | |  |
| Male | 172 (52) | 19556 (42) | 78 (59) | | 8846 (41) | 85 (55) | | 12666 (43) | 90 (50) | | 12434 (42) |
| Female | 156 (48) | 26846 (58) | 55 (41) | | 12730 (59) | 70 (45) | | 16921 (57) | 89 (50) | | 17283 (58) |
| **Side** n (*%*) |  |  |  | |  |  | |  |  | |  |
| Right | 173 (53) | 24089 (52) | 112 (84) | | 18701 (87) | 71 (46) | | 15425 (52) | 88 (49) | | 15492 (52) |
| Left | 155 (47) | 22313 (48) | 21 (16) | | 2875 (13) | 84 (54) | | 14162 (48) | 91 (51) | | 14225 (48) |
| **ASA classification**  n (*%*) |  |  |  | |  |  | |  |  | |  |
| 1 | 59 (18) | 7505 (17) | 21 (16) | | 3628 (17) | 27 (17) | | 5217 (19) | 34 (19) | | 5086 (18) |
| 2 | 209 (64) | 30054 (68) | 90 (68) | | 14211 (69) | 96 (62) | | 19056 (67) | 107 (60) | | 19044 (68) |
| 3 | 60 (18) | 6728 (15) | 22 (16) | | 2844 (14) | 32 (21) | | 4061 (14) | 38 (21) | | 3991 (14) |
| 4 | 0 (0) | 43 (0) | 0 (0) | | 15 (0) | 0 (0) | | 22 (0) | 0 (0) | | 19 (0) |
| 5 | 0 (0) | 1 (0) | 0 (0) | | 0 (0) | 0 (0) | | 0 (0) | 0 (0) | | 0 (0) |
| **Type of articulation**  n (*%*) |  |  |  | |  |  | |  |  | |  |
| CR | 248 (77) | 43697 (95) | 102 (78) | | 20403 (95) | 121 (80) | | 28053 (95) | 143 (81) | | 28264 (95) |
| PS | 46 (14) | 2047 (4) | 18 (14) | | 863 (4) | 17 (11) | | 1123 (4) | 21 (12) | | 1062 (4) |
| CCK | 17 (5) | 419 (1) | 4 (3) | | 213 (1) | 10 (6) | | 308 (1) | 8 (4) | | 277 (1) |
| Hinged | 13 (4) | 113 (0) | 7 (5) | | 39 (0) | 4 (3) | | 33 (0) | 5 (3) | | 54 (0) |
| **Year of TKA** n (*%*) |  |  |  | |  |  | |  |  | |  |
| 2000 – 2015 | 76 (23) | 13895 (30) | 29 (22) | | 6321 (29) | 58 (37) | | 10850 (37) | 67 (37) | | 12219 (41) |
| 2016 – 2021 | 252 (77) | 32507 (70) | 104 (78) | | 15255 (71) | 97 (63) | | 18737 (63) | 112 (63) | | 17498 (59) |
| Data are presented as mean (standard deviation) or n (%). In the unmatched cohorts, category totals for some variables do not sum to the total cohort size because some data were missing in the registry. *BMI,* body mass index*; ASA,* American Society of Anaesthesiologists*; PTOF,* posttraumatic osteoarthritis after knee fracture osteosynthesis*; OA,* knee osteoarthritis without prior significant knee surgery or fractures*; TKA,* total knee arthroplasty; *KOOS-12*, a 12-item short form of the Knee injury and Osteoarthritis Outcome Score; *EQ-5D-3L*, the 3-level version of EQ-5D, a standardised measure of health-related quality of life developed by the EuroQol Group; *CR*, cruciate retaining; *PS*, posterior stabilized; *CCK*, constrained condylar knee. | | | | | | | | | | | |

| **Supplementary Table S2. Sensitivity analysis of 1-year postoperative Satisfaction and Pain Likert scales stratified by type of knee articulation** | | | |
| --- | --- | --- | --- |
| **Stratified analysis by the type of articulation – 1-year postoperative Satisfaction Likert scale** | | | |
|  | **PTOF group** | **OA group** | **P-value** |
|  | **Number (%)** | **Number (%)** |  |
| **CR** | | | |
| 1. Very dissatisfied | 6 (*2.5*) | 7 (*1.2*) | **0.012 *** |
| 1. Dissatisfied | 20 (*8.3*) | 23 (*4*) |  |
| 1. Neither nor | 31 (*13*) | 52 (*9.1*) |  |
| 1. Satisfied | 77 (*32*) | 179 (*31*) |  |
| 1. Very satisfied | 108 (*45*) | 311 (*54*) |  |
| Total | 242 (*100*) | 572 (*100*) |  |
| **PS** | | | |
| 1. Very dissatisfied | 2 (*4.7*) | 1 (*4.5*) | **0.9 *** |
| 1. Dissatisfied | 3 (*7*) | 3 (*13*) |  |
| 1. Neither nor | 7 (*16*) | 3 (*13*) |  |
| 1. Satisfied | 17 (*39*) | 7 (*32*) |  |
| 1. Very satisfied | 14 (*32*) | 8 (*36*) |  |
| Total | 43 (*100*) | 22 (*100*) |  |
| **CCK** | | | |
| 1. Very dissatisfied | 1 (*5.9*) | 0 (*0*) | 0.799 ***** |
| 1. Dissatisfied | 1 (*5.9*) | 0 (*0*) |  |
| 1. Neither nor | 4 (*23*) | 2 (*20*) |  |
| 1. Satisfied | 3 (*17*) | 3 (*30*) |  |
| 1. Very satisfied | 8 (*47*) | 5 (*50*) |  |
| Total | 17 (*100*) | 10 (*100*) |  |
| **Hinged** | | | |
| 1. Very dissatisfied | 0 (*0*) | 0 (*0*) | 1 ****** |
| 1. Dissatisfied | 0 (*0*) | 0 (*0*) |  |
| 1. Neither nor | 0 (*0*) | 0 (*0*) |  |
| 1. Satisfied | 1 (*50*) | 1 (*25*) |  |
| 1. Very satisfied | 1 (*50*) | 3 (*75*) |  |
| Total | 2 (*100*) | 4 (*100*) |  |
| **Stratified analysis by the type of articulation – 1-year postoperative Pain Likert scale** | | | |
|  | **PTOF group ^£^** | **OA group** | **P-value** |
|  | **Number (%)** | **Number (%)** |  |
| **CR** | | | |
| 1. None | 24 (*24*) | 94 (*40*) | **0.003 *** |
| 1. Very mild | 27 (*27*) | 71 (*30*) |  |
| 1. Mild | 27 (*27*) | 36 (*15*) |  |
| 1. Moderate | 15 (*15*) | 24 (*10*) |  |
| 1. Severe | 7 (*7*) | 5 (*2.2*) |  |
| Total | 100 (*100*) | 230 (*100*) |  |
| **PS** | | | |
| 1. None | 6 (*35*) | 6 (*50*) | 0.487 ***** |
| 1. Very mild | 6 (*35*) | 4 (*33*) |  |
| 1. Mild | 1 (*5.9*) | 0 (*0*) |  |
| 1. Moderate | 4 (*23*) | 1 (*8.3*) |  |
| 1. Severe | 0 (*0*) | 1 (*8.3*) |  |
| Total | 17 (*100*) | 12 (*100*) |  |
| * Chi square test, ** Fisher’s exact test  *PTOF*, posttraumatic osteoarthritis after knee fracture osteosynthesis*; OA,* knee osteoarthritis without prior significant knee surgery or fractures*; CR*, cruciate retaining; *PS*, posterior stabilized; *CCK*, constrained condylar knee.  **^£^** There were 4 fewer PTOF cases in the stratified analysis than in the overall Pain Likert cohort because 4 PTOF cases with a CCK knee did not have an OA match and had to be excluded. | | | |

| **Supplementary Table S3. Availability and overlap of PROM data across PROM-specific matched cohorts** | | | | | |
| --- | --- | --- | --- | --- | --- |
|  | **Satisfaction PROMs** | **Pain PROMs** | **KOOS-12 PROMs** | **EQ-5D-3L PROMs** | **Total** |
| **PTOF cases after matching** | | | | | |
| **Satisfaction cohort** | 304 (100) | 121 (40) | 137 (45) | 160 (53) | 304 |
| **Pain cohort** | 121 (100) | 121 (100) | 81 (67) | 77 (64) | 121 |
| **KOOS-12 cohort** | 137 (97) | 81 (57) | 141 (100) | 102 (72) | 141 |
| **EQ-5D-3L cohort** | 160 (98) | 77 (47) | 102 (63) | 163 (100) | 163 |
| **Controls after matching** | | | | | |
| **Satisfaction cohort** | 608 (100) | 12 (2) | 25 (4) | 31 (5) | 608 |
| **Pain cohort** | 12 (5) | 242 (100) | 23 (10) | 37 (15) | 242 |
| **KOOS-12 cohort** | 25 (9) | 23 (8) | 282 (100) | 41 (15) | 282 |
| **EQ-5D-3L cohort** | 31 (10) | 37 (11) | 41 (13) | 326 (100) | 326 |
| Data are presented as n (%). Collums show the number of TKAs with overlapping PROMs for the specific PROM cohort in each row. Cells in the last column indicate the total size of each PROM-specific cohort. *PTOF*, posttraumatic osteoarthritis after knee fracture osteosynthesis*; OA,* knee osteoarthritis without prior significant knee surgery or fractures*; KOOS-12*, a 12-item short form of the Knee injury and Osteoarthritis Outcome Score; *EQ-5D-3L*, the 3-level version of EQ-5D is a standardised measure of health-related quality of life developed by the EuroQol Group | | | | | |
